# Improving the relational aspects of trauma care through translational simulation

**DOI:** 10.1186/s41077-019-0100-2

**Published:** 2019-05-21

**Authors:** Victoria Brazil, Eve Purdy, Charlotte Alexander, Jack Matulich

**Affiliations:** 1 Faculty of Health Science and Medicine, Bond University, Robina, QLD Australia; 2 Gold Coast Hospital and Health Service, Southport, QLD Australia; 3 Queen’s University, Kingston, ON Canada; 4 Emergency Department, Gold Coast Hospital and Health Service, Southport, QLD Australia; 5 Simulation Service, Gold Coast Hospital and Health Service, Southport, QLD Australia

**Keywords:** Teamwork, Trauma care, Simulation, Ethnography, Culture

## Abstract

**Background:**

Major trauma care is complex and requires individuals and teams to perform together in time critical, high-stakes situations. Scenario-based simulation is well established as a strategy for trauma teamwork improvement, but its role in the relational and cultural aspects of trauma care is less well understood. Relational coordination theory offers a framework through which we aimed to understand the impact of an established trauma simulation programme.

**Methods:**

We studied simulation activities using a narrative survey of trauma providers from anaesthesia, emergency medicine, medical imaging, surgery, trauma service, intensive care, and pre-hospital providers at Gold Coast University Hospital, in conjunction with data from an ethnography. Data analysis was performed using a recursive approach—a simultaneous deductive approach using the relational coordination framework and an inductive analysis.

**Results:**

Ninety-five of 480 (19.8%) staff completed free-text survey questions on simulation. Deductive analysis of data from these narrative survey results using the RC framework domains identified examples of shared goals, shared knowledge, communication and mutual respect. Two major themes from the inductive analysis—“Behaviour, process and system change” and “Culture and relationships”—aligned closely with findings from the RC analysis, with additional themes of “Personal and team learning” and the “Impact of the simulation experience” identified.

**Conclusions:**

Our findings suggest that an established trauma simulation programme can have a profound impact on the relational aspects of care and the development of a collaborative culture, with perceived tangible impacts on teamwork behaviours and institutional systems and processes. The RC framework—shared knowledge, shared goals and mutual respect in the context of communication that is timely, accurate, frequent and problem-solving based—can provide a common language for simulation educators to design and debrief simulation exercises that aim to have a translational impact.

## Background

*Major trauma care* is complex and requires individuals and teams to perform together in time critical, high-stakes situations. Effective trauma systems, individual provider skills and teamwork behaviours are required for good patient outcomes [1] and are frequently targeted for improvement efforts, including the use of simulation [2–5]. Evaluation of simulation interventions in trauma has been largely focused on skills or knowledge acquisition, although teamwork behaviours and clinical outcomes are emerging measures [6, 7]. These effective teamwork behaviours and trauma care systems are technical, observable and supported by evidence but are arguably underpinned by institutional culture and relationship*s* between individuals and groups involved [8]. The links between organisational culture and healthcare performance are complex [9], but there appear to be tangible differences between high- and low-performing institutions [10]. The impact of simulation interventions on the relational aspects of care, including culture, is not well documented, despite an intuitive and theoretical appeal [11].

*Translational simulation* [12] in healthcare is focused directly on health service priorities and patient outcomes and offers a framework for how trauma care may be improved through the *interventional* and *diagnostic* functions of simulation programmes. Recent enthusiasm for in situ simulation—simulation delivered in the real clinical environment—has supported teamwork training, and the identification of safety threats related to the environment or care systems within that real clinical environment [4, 13–15], and offers the opportunity for enhanced translational impact. However, most in situ simulation design remains focused on the clinical conditions being simulated, rather than the design and debriefing strategies to enhance team interfaces, institutional culture and relational aspects of care. Simulation programme leaders have limited guidance when focusing on these goals.

Understanding the effects of a specific intervention, such as simulation, on culture is not simple. Traditionally, anthropologists engage in ethnography, embedded participant-observation over an extended period of time, to understand the rituals, values, beliefs, behaviours, relationships and practices of a specific group. Traditional ethnography with an inductive approach allows for broad understanding of culture, but the application of recognised theories in this context can also be helpful when approaching specific questions. Insights gained through ethnographic study need not be strictly inductive or deductive in nature but rather fall along the spectrum from discovery to validation [16].

**Fig. 1:**
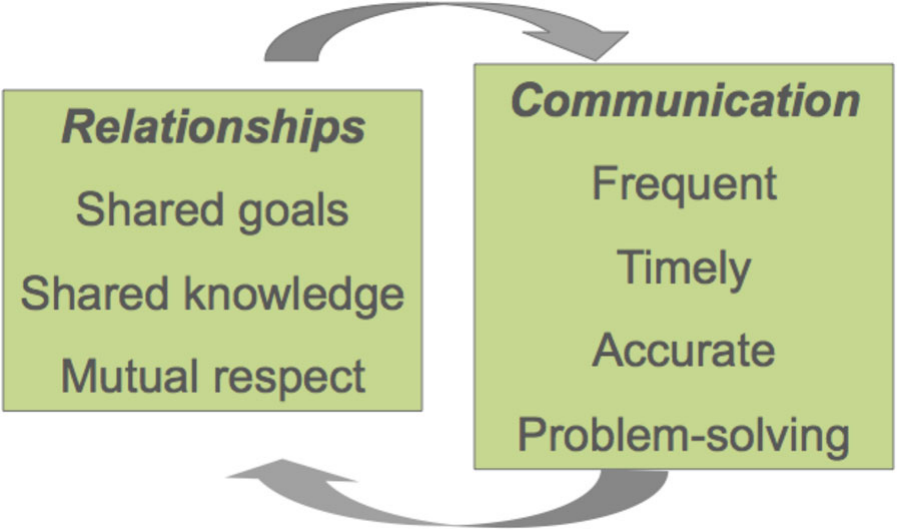
Relational coordination framework

*Relational coordination* (*RC*) theory [17] was originally developed in business but has been applied across multiple healthcare contexts [18, 19] to understand the relational dynamics of coordinating work between groups in organisations. The framework (Fig. 1) specifies three attributes of relationships that support the highest levels of coordination and performance—*shared goals* that transcend participants’ specific functional goals, *shared knowledge* that enables participants to see how their specific tasks interrelate with the whole process and *mutual respect* that enables participants to overcome the status barriers that might otherwise prevent them from seeing and taking account of the work of others. (10) The three relational dimensions of RC are reinforced by specific aspects of communication that support coordination and high performance, namely *frequency*, *timeliness*, *accuracy* and, when problems arise, a focus on *problem-solving* rather than blaming. [20] Pairing a traditional ethnography with an application of the RC framework provides an opportunity to understand how simulation fits into the broader culture of trauma care provision, while also revealing sufficient granularity in specific relational domains to provide practical guidance for simulation educators.

We aimed to understand how an established trauma simulation programme is perceived by trauma care providers to influence their relationships with others and to identify those aspects of the simulation experience contributing to relational outcomes.

## Methods

### Study setting

The Gold Coast University Hospital (GCUH) established a trauma service in 2013, concurrent with the opening of a new physical facility. Over 400 staff from a variety of disciplines and work groups participate in the care of major trauma patients in the hospital. In the financial year 2017–2018, there were 1739 trauma team activations, including 203 “Trauma Responds”, which is the highest acuity level at the institution.

**Fig. 2:**
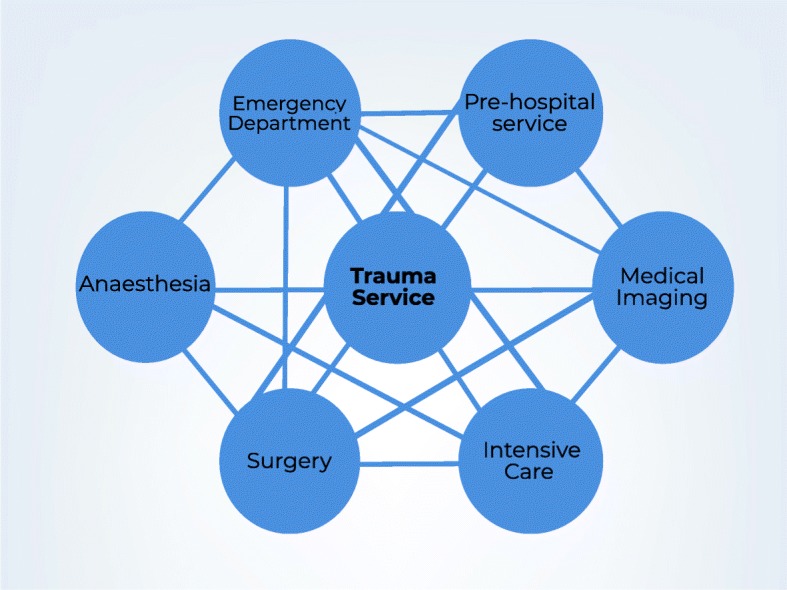
Groups involved in trauma care

There are key identifiable groups involved in the early phases of major trauma care and their inter-relationships for the initial phases of major trauma patient care are represented in Fig. 2. Each group is represented by function and/or geographic location, rather than professional discipline.

### Trauma simulations

Over the last 4 years, the trauma and simulation services at GCUH have collaborated to improve trauma care at the hospital through monthly in situ simulation of trauma cases. These simulations are conducted involving providers from across the care continuum—paramedics, emergency department (ED) staff, medical imaging, operating theatre staff, surgery teams and intensive care teams, as well as support services such as blood bank, orderlies and security. These provider groups have voluntarily elected to join the trauma simulation process over that time. Staff are expected to participate in these simulations as part of their education, and as a standard part of trauma service delivery improvement.

Trauma simulations are scheduled at a consistent time each month. Participating staff are aware of simulation in advance and are emailed written preparatory information, including the broad clinical issues that will be the focus. Scenarios are designed based on common and important trauma presentations drawn from GCUH trauma database. Scenarios are presented using both manikin and simulated patient methodologies, according to the case and challenges designed. A 5–10-min pre-briefing is conducted in a conference room, followed by a 45-min scenario conducted in situ—initially in the ED ambulance bay and trauma room, and in some cases followed by transfer to computed tomography (CT) scanner or operating theatre. Between 8 and 30 staff participate in these simulations, with up to another 40 staff observing via video feed in the conference room, where the subsequent debrief is conducted for a further 30–45 min. The debrief is led by an experienced simulation debriefer who is also a senior clinician within the institution.

The large group debrief following the simulations are targeted towards practitioners involved in the simulated patient journey but with participation from observers also encouraged. The facilitator prompts reflection on the clinical processes and outcomes and enables discussion between providers to identify problems and proposed solutions. The debrief issues are summarised after each simulation in a quality activity report, provided to service leads from each department involved in the simulation. More recently, learning points have also been summarised in infographic format, which is then circulated to staff from the departments involved in trauma care.

### Study design and data collection

As one aspect of a multi-phase quality improvement project aimed at understanding and supporting relationships between trauma care providers at our institution, we studied ongoing trauma simulation activities using a narrative survey in conjunction with data from a broader ethnography of the trauma care provision, which naturally included data related to simulation activities. The focused look at simulation reported here was performed in the context of a more extensive evaluation of how relationships affect the coordination of clinical trauma care at GCUH.

**Fig. 3:**
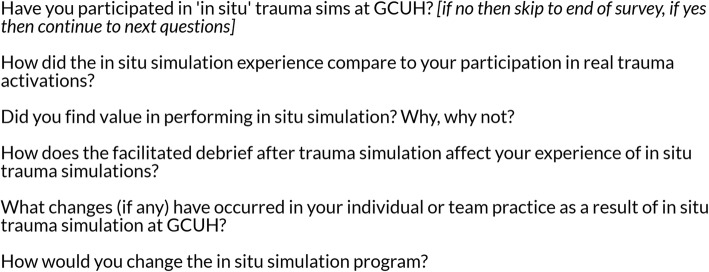
Survey questions

Staff from the key areas involved in the early phase of major trauma care at GCUH—emergency, trauma service, surgery, medical imaging, intensive care unit, paramedics and anaesthetics—were invited to participate. A link was provided via email to a survey that included 5 free-text questions related to their experience of in situ simulation in trauma care at GCUH (Fig. 3).

These narrative questions were one element of a survey that also included a quantitative relational coordination (RC) survey [17] comprising of Likert item questions related to the respondents’ perceptions of relationships between provider groups in trauma care at GCUH. Data from this quantitative part of the survey was not specifically related to simulation activities and is not reported here. Completion of the survey was voluntary and anonymous.

Concurrently, an ethnography of the trauma team and simulation programme was performed by author EP over a 3-month period. She conducted participant-observation of real and simulated traumas, educational activities of the trauma team, and the day-to-day activities of members from the core working groups for a total of approximately 75 h of participant-observation (16 h directly related to trauma simulations) and an additional 25 h of informal interviewing. [21] These activities informed field notes through a process similar to that described by Schensul and LeCompte [21]. Five formal interviews were performed with key personnel to further explore issues raised in the survey or through observation. Interviews were recorded and transcribed. Data from field notes, informal, and formal interviews generated from this process that related to trauma simulations are included in this review.

### Data analysis

Data analysis was performed using a recursive approach, a simultaneous deductive and inductive approach to data interpretation. Data from the surveys, field notes and interviews was entered into NVivo software then coded by two authors (EP and CA) using the relational coordination framework (knowledge, goals, respect and communication), themes not accounted for by this framework were generated inductively. An independent inductive analysis of the free-text questions related to trauma simulation was also performed by VB and JM. All authors then met to compare findings and create agreement on relevant themes and subthemes. The association between the relational coordination themes and inductive interpretation of findings was compared and contrasted by authors to finalise the interpretative analysis presented.

### Notes on the research team

Victoria Brazil (VB) is an emergency physician and simulation educator. She has led the trauma simulation programme at the institution since its inception and facilitates most of the debriefing sessions after trauma simulations. She participates as a trauma team leader at the institution in her role as an emergency physician. Eve Purdy (EP) is a master’s student in applied anthropology and an emergency medicine trainee from Canada, currently undertaking fellowship year at the institution. She has led the ethnographic component of a group of research projects focused on trauma care and emergency medicine at GCUH. She also works as a trauma team member. Charlotte Alexander (CA) is a provisional trainee in emergency medicine at the institution, and occasional trauma team member. She has been involved in the trauma simulations since 2014 as an assistant simulation educator, while still a medical student at Bond University. Jack Matulich (JM) is a registered nurse, currently working within the GCUH Simulation Service, who has been involved in delivery of trauma simulations over the last 6 months. JM and CA were both trained and received guidance in the analytical methods used in this study by EP.

The broader research team comprised an advisory group with members drawn from pre-hospital service, emergency medicine, anaesthesia, intensive care, medical imaging and the trauma service.

Consultant physicians, simulation team members and nurses outside of the research team, but familiar with the simulation exercises, were consulted for a member check of the findings.

## Results

Four hundred and eighty staff, from all groups involved in trauma care, were invited to participate in the overall RC and simulation survey. One hundred and eighty responses were received (38%). One hundred of 180 (56%) indicated they had been involved in trauma simulations, and 95 completed the survey questions on simulation. Respondents were from all departments involved in the survey.

**Table 1:** Participant perceptions of Trauma Simulations

Theme	Subtheme	Representative examples
Behaviour, process and systems change		
	Communication & teamwork behaviours	Good to practice complex competing head injury with shock and other organ/systems and sort out priorities and to further develop improved ways of managing multiple simultaneous tasks including blood product resuscitation.
		Creation of realistic Sim gives the opportunity to develop and standardise handover and assessment/interventions procedures in a realistic manner.
		High value in terms of team training, process training and exercise of new protocols, exercise of new communication practices (handover in OT of red blanket patient), quite high fidelity training even of technical skills!
	Role allocation and team structures	I [now] try to get everyone’s name at the start of the day and make an effort to illustrate the whole teams concerns about a case before we start
		More focus on a standardised process, clearer communication, avoiding “loading up” the TTL.
		I have become more familiar with the actual roles and workload of various other parties/individuals in the trauma team.
		Delegating a task to a micro team and allowing their full autonomy unless there is an issue (airway/transfusion protocol)
	Leadership	(Now) very clearly signposting verbally to the team the most important priority setting verbal times for when things need to happen by—“It is 3 O’clock now, we are aiming to be on our way to CT at 3:10”.
		Increased awareness of the value of “stepping back” as a team leader without hands on the patient.
	Process review and improvement	Provided the opportunity to think through the processes and also where to go and who to talk to etc. especially when we took pt. straight the theatre which is somewhere I have not been before. We have introduced procedures such as the pre and post.
		Intubation checklist, and had the opportunity to try different variations of this. We also have had the opportunity to fine tune procedures and test communication styles.
		We have been provided access to the Resus camera’s so we can monitor progress of the patients arrival to CT.
Culture and relationships		
	Interdisciplinary role understanding	Very effective in highlighting the subtle differences in priorities between the ED, Surgical, ICU and Anaesthetic teams when managing a patient.
		An opportunity to practice, the focus on processes and communications, getting to know the other team members in a learning environment, getting a better sense of the roles of others and the pressures/challenges that involves (esp. nursing).
		Chance to gauge other people’s opinion and point of view of the trauma. Great opportunity to promote dialogue amongst different specialties.
		There is a big role for ED staff to orientate the specialist teams and define anticipated tasks and where their help is required.
	Team building	I think the real value is getting to know our colleagues from other departments outside of a real life stressful situation. That familiarity is then incredibly helpful when faced with a real trauma.
		There is a culture of mutual respect between the specialty teams involved which is fostered by the debrief and carries over into the real world.
		Mostly it promotes team-building, both in-house and between specialties.
		My practice remains the same, though I have noticed inter-departmental relationships are better as a result of the sims.
Personal and team learning		
	Personal and team reflection process and habit	Because we practise together becoming like a team that is being coached. We get to know each other, give space and gain understanding of each other’s roles, and are informed and reminded of our common goals. (time to protect airway, time to OT) and common challenges (safety in CT scanner with unstable patient).
		Often are we make some solid learning points and debrief areas of improvement. I think everyone is getting better at useful discussion and not blame.
		Made me look at my communication style and adjust how I interacted with others in a real situation.
		Definitely helps to reflect on good/bad things and work towards solutions as a group.
	Personal learning, confidence & motivation	My own personal learning of the systems down in ED, making connections to faces, and being able to possibly improve on areas in anaesthetic related care in another’s department.
		I have had sim cases that I have not dealt with before in real cases. This sparks an interest in the situation and encourages adaption to whatever scenario may present.
		Help organise and focus my own thinking and processing of information on how we manage the patient journey.
		I find that the debrief afterwards is always such an goldmine for the technical points but also for the non-technical skills in thinking of the things that could go wrong before they do.
		We all get nervous and it’s a great way of stepping through situations that you could possibly be faced with hours after the sim, it helps you prepare mentally.
		Makes me brush up on certain detailed knowledge that perhaps I did not have at my instant recall - invites me to continue to read around cases and improve.
	Translation to practice	I have become more familiar with equipment in the emergency department and am therefore less likely to rely on equipment I bring from my own environment.
		I have found that those who participate in the sims incorporate the lessons into their practice, especially around communication and collaborative decision making.
		I have noticed improvements over the years because of this both in my handovers and the reception of them. Knowing what the ED staff needs allows me to better streamline and deliver handovers to them.
		Many (*changes*). I communicate in resus differently now. I advocate for names on gowns. The TS nursing staff try to take on the transfusion role in resus which is new and where we are most useful.
		We have changed processes, moved to a focused hands off QAS handover, recognised and adapted communication issues and fears in foreign environments. Noted strengths in staff not previously recognised such as planning and predictive actions taken by experienced porterage staff.
The impact of the simulation experience		
	Perceptions of realism related to stress/emotional fidelity, and team/task fidelity	It felt legitimate and people seemed to take it very seriously—the more complex and stressful the pt. the more the team treats it as a real situation.
		High level of realism with elevating noise levels, multiple individuals involved and complex disposition planning.
		The in situ simulation is very realistic to the real situations. They are usually noisy events with lots of people you probably have not met before. They do re-create the real world issues we face with managing the team, trying to not lose momentum etc.
		Very well! Staff exhibited many of the same emotions and work stresses associated with real cases. Many minor issues arose in the Sim that replicated itself in actual cases.
		Pretty similar to DAY TIME traumas. Totally different to night time traumas.
	Experimentation/learning supported by lack of perceived risk to patients	More time to see process from others’ perspectives; non-stressful and non-threatening environment which encourages learning.
		Yes—debriefing encourages different departments to communicate in ways that cannot occur when a sick pt. still needs active management in a structured and led safe space.
	Facilitated debrief creating safe space for discussion/disclosure/reflection	Often if you are in the sim you may have a biased perspective, i.e. you think you did worse or better than you actually did so debrief helps to get outside perspective on performance and if any improvement could be present.
		Good to get other speciality feedback from a non Ed perspective as we do not often get to discuss these issues in a constructive environment.
		Often walk away feeling less “judged” than I always anticipate.
		I find the experience empowering, as you are afforded the opportunity to discuss things in a calmer environment. You can receive positive feedback which makes you feel good. It’s not an environment of “name and shame”. We are lucky enough to have a decent pool of people who run and debrief the scenarios.
		Makes it feel safe to participate again because I feel very safe with debriefers. They are very good at pointing out how we can improve as a team and making common goals more clearly visible.

### Relational coordination analysis

During deductive analysis using the RC framework all domains of the construct were easily identified. The concept of simulation as an enabler of mutual respect was most predominant followed closely by its effect on the development of high-quality communication. The ability for simulation to facilitate shared goals and shared knowledge were less prominent but also readily recognised. We review each of these themes below.

In terms of shared goals, participants commented on how the simulation exercises allowed them to understand common shared management priorities:

[the simulations are] Absolutely of value … We practice together, becoming like a team that is being coached. We get to know each other, give space and gain understanding of each other’s roles, and are informed and reminded of our common goals (time to protect airway, time to OT [operating theatre]) and common challenges (safety in CT scanner with an unstable patient). – survey respondent

Some participants reflected on how practice in simulations translated to achievement of shared goals in clinical work. For example, one defined goal of the trauma service is the seamless transition of patients between services – prehospital, to the operating theatre, and between teams. A paramedic noted how simulations have improved his own handover process.

I have noticed improvements over the years because of this [simulation] both in my handovers and the reception of them. Knowing what the ED staff needs allows me to better streamline and deliver handovers to them. – survey respondent

Overall, we found that participants saw simulation both as a way to identify shared goals as a group, then practise and troubleshoot movement towards them.

In the domain of shared knowledge, two distinct types of knowledge were identified, clinical knowledge related to the specifics of the simulated case and knowledge of trauma roles and processes. One nurse commented, “as a nurse it consolidates my understanding of the injuries and treatment and where things went wrong or right and why” (survey respondent). However, thoughts related to specific clinical practice were less common than comments describing improved knowledge of the roles and processes of the trauma team. One respondent noted, “I have become more familiar with the actual roles and workload of various other parties/individuals in the trauma team” (survey respondent). Another reflected that the value of simulation was related to understanding the differences between ED and anaesthetic airway management and the department specific protocols unique to the ED environment.

Awareness of ED protocols (e.g. the airway checklist) and how this can be acknowledged by us (anaesthetics) without compromising safe and timely airway intervention. – survey respondent

Mutual respect was the most commonly inductively coded domain in both the survey data and field notes. Phrases such as “breaking down barriers”, “team building” and “connection” were frequently used by participants to describe why they felt the simulation exercises were valuable. Some participants clearly outlined how simulations impact professional relationships.

I think the real value is getting to know our colleagues from other departments outside of a real-life stressful situation. That familiarity is then incredibly helpful when faced with a real trauma. – survey respondent

[The team leader] also reflected on the fact that it was a bit challenging to work with people that you don’t know. He said, “at the old hospital you just called up and they were your ‘mates’ but that’s not the case here”. Later in the debrief both the neurosurgeon and the ICU consultant reflected on the fact that this was a nice way to actually make some of those relationships and get to know each other. – field notes

In the final domain, communication, there were many generic comments such as, “I think it gives you a good chance to work on your communication skills with colleagues” (survey respondent). However, the relational coordination framework further divides communication down into four component parts (frequent, timely, accurate and problem-solving based). When comments were detailed enough for this analysis, we found that participants reflected most-often on the problem-solving-based aspect of communication. They identified simulation as a place to practise collaborative decision-making between specialties with one participant commenting, “I have found that those who participate in the sims incorporate the lessons into their practice, especially around communication and collaborative decision making” [survey respondent]. Consistent focus on accurate communication methods was noted as translating into practice by some participants in the survey and informal interviews, with one commenting, “communication has greatly improved across our trauma team. clear role allocation early, clear leader, close loop communication, etc.” (survey respondent). Comments related to frequent and timely communication were rarely identified; however, informal discussions and observations throughout the ethnographic process facilitated understanding that the simulation exercise itself serves to promote frequent communication—not only around a specific patient but as a system—by providing a space each month for specialties to come together for open dialogue. That facilitated space seemed to foster a frequent, respectful and problem-solving-based interdisciplinary conversation.

### Inductive analysis

The inductive analyses of survey responses supported and extended the findings of analysis guided by the RC framework and are presented in Table 1. We identified four major themes: “Behaviour, process and system change”, “Culture and relationships”, “Personal and team learning” and “Impact of the simulation experience”.

Two of the major themes from the inductive analysis—*Behaviour, process and system change* and *Culture and relationships*—aligned closely with findings from the RC analysis. Tangible changes and improvements were noted in wide-ranging practical examples across professions and departments. These changes ranged from modest, incremental improvements (e.g. checklists and handovers) but also included fundamental team structure concepts related to sub-team autonomy and team leader cognitive load as alluded to in field notes from the debrief after a simulation of a patient with competing abdominal and neurotrauma priorities.

“There was general consensus that assigning ourselves into functional teams (airway, breathing, circulation, procedures, external management) may be better than organizing ourselves by our disciplines (anaesthetics, nursing, ED, surgery). However, many reflected that we feel more comfortable viewing ourselves by our disciplines because that is the way that we have been trained and those are the people who we recognize. We got to the point that we decided that in this type of complex situation sub teams are probably a good idea, and started to talk explicitly about how to make that happen. – field notes

Additional themes that were identified in the inductive analysis were *Personal and team learning* and the *Impact of the simulation experience.* The habits of shared reflection seemed to be as important as the learning from any specific simulation experience. As with the domain of shared knowledge from the RC framework, the personal and team learning related to both “technical” trauma care lessons, and those relating to team roles, functions and relationships. Many practical examples of translation to practice focused on communication and other teamwork behaviours, with relatively few relating to technical skills.

Respondents perceived the simulation experience (and its design, delivery and debriefing) to be relevant to the outcomes. The need for a high degree of task realism was clear, including technical and environmental aspects but even more important was the degree of realism of interactions, challenges and affective experience. The absence of comments relating to lack of realism of manikin was notable.

Both the RC and inductive framework were supported by local and more broad member checks, and no new themes were identified.

## Discussion

Our findings suggest that an established trauma simulation programme can have a profound impact on the relational aspects of care and the development of a collaborative culture, with perceived tangible impacts on teamwork behaviours and institutional systems and processes. This effect was observed in simulations with a perceived high degree of team and task realism, and with explicit simulation design, preparation and debriefing approach targeted towards relational aspects of care. To what extent the impact is attributable to each individual factor is not known, but overall, study respondents recognise a significant impact of the simulation experience on their learning and development of relationships with other care providers and teams.

Over half of trauma providers who answered the overall RC survey had participated in trauma simulations, evidence of significant engagement of these provider groups with the simulation process. The extent to which this level of engagement, over a sustained period, is required for this impact is also not known nor is whether that engagement is cause or effect of the relational impact and developing collaborative culture. As Mannion et al. point out “any relations between culture and health service outcomes are likely to be mutual and recursive: that is, perceived performance is as likely to shape local healthcare cultures as culture is to shape local healthcare performance” [9].

The straightforward application of the RC framework to the simulation experience data, and strong alignment of inductively generated results with the RC framework, provide compelling evidence that simulation has the ability to target and bolster fundamental domains of high functioning teams—shared knowledge, shared goals and mutual respect in addition to high-quality communication. These relational outcomes are stepping stones towards an improvement in organisational culture and go below the surface of previously documented benefits of simulation [14, 15, 22]. Furthermore, our results provide support for application of RC theory to inform the design, delivery and debriefing of simulation activities focused on achieving relational goals within clinical trauma care practice.

The findings specific to the simulation experience are instructive. The subtheme “facilitated debrief creating safe space for discussion/ disclosure/ reflection” strongly supports the concept of psychological safety “as an emergent property of the collective, that describes the level of interpersonal safety experienced by people in a particular group” [23]. Simulation educators have focused on establishing psychological safety for simulation activities [24], and our findings raise the possibility of translation into clinical practice—such that a simulation programme, through enhancing relationships and fostering mutual respect, can support the development of psychological safety for providers beyond the simulation exercise and positively affect real clinical practice.

With respect to practical simulation delivery, there was broad agreement on the high degree of realism within the simulations. Consistent with recent literature, the basis of that realism was rarely cited as physical resemblance and more commonly related to functional task alignment [25]. As such, translational simulation applied to a trauma patient journey with involvement of diverse provider groups presents a design challenge—being able to print an ECG may be a key element of realism for one staff member, while another requires a high degree of realistic stress as a trauma team leader.

### What this means for trauma providers and clinical leaders

Simulation should be considered as a tool to build and strengthen relationships between practitioners across traditional boundaries. A dedicated trauma simulation programme may offer wide-ranging opportunities to improve culture and relationships that are difficult to approach using other strategies. Health professionals providing trauma care perceive that trauma simulations affect relationships and culture, and that this translates to real-world practice. The learning from simulation is multi-faceted—including motivation, personal leadership, teamwork and communication behaviours, and local systems and protocols. Regular simulation affords practitioners the space to come together in an environment that stimulates the habits of reflection for both individuals and groups. Our participants suggest that this has an impact that extends well beyond simulation sessions.

### What this means for simulation providers

The RC framework can be applied to the acute trauma setting, not only as a diagnostic tool for measuring team function as traditionally designed but also as a pillar for guiding the development of translational simulation interventions and structuring debriefing conversations for translational impact. Developing recognisable language to describe relational fundamentals in debriefing conversations—shared goals, shared knowledge and mutual respect—can complement existing language related to observable teamwork behaviours.

Simulation providers need to carefully support task realism for all provider roles involved in trauma simulations, including technical task alignment but also create realistic affective experience and team interactions. Having intact teams participate in trauma simulations, including leadership by senior clinicians, is an opportunity afforded more easily by using in situ delivery.

### What this means for researchers

The novel application of the RC framework to analyse the impact of simulation can serve as an example for others interested in examining the role of simulation in affecting relationships and culture at their institution. We found that pairing the narrative surveys with an ethnography, to be uniquely informative in understanding the role of simulation within the overall trauma service and would encourage consideration of this embedded approach. As with many ethnographies, we are left with more questions than answers. Some relate to examining the “dose” of translational simulation required to have a relational impact, while others centre on understanding the granular elements of simulation design, delivery and debriefing that lead to relational outcomes.

### Limitations

The most significant limitation of this work is that the effects of any simulation programme will be dependent on a number of factors including local context, pre-existing relationships, design and delivery. The findings we present are local to our institution and may not translate to other programmes already in existence or developed in the future. Our results show simulation driven movement towards positive relational outcomes but the opposite could also occur under different circumstances. We trust that simulation educators will apply our method, not just focus on our results, in an effort to thoughtfully consider the effect of their local programmes on culture and relationships.

Next, the respondents answered survey questions related to trauma simulations after completing the overall RC survey, which included questions focused on their relationships with other trauma providers. This may have primed respondents towards responses focused on relational aspects of care. However, this bias is simply towards the topic of relationships—the extent of positive relational impact and more collaborative culture stands independently. Furthermore, including a less bounded question about individuals’ experiences with trauma simulation might have added to our understanding.

Respondents may have undertaken their simulation experience at any time within the 4 years preceding the survey, and their experience may have been as few as one simulation or as many as 25. These results should therefore be regarded as a review of the programme, rather than any one specific simulation activity.

Finally, the study authors and simulation providers have been focused on relational aspects of care in the design and delivery of trauma simulations, and acknowledge they may be inclined towards interpretation of survey results and field notes in the light of these interests. However, the strength and consistency of those themes and enthusiastic support for the findings in member checks is suggestive that the findings stand on their own.

## Conclusion

An established trauma simulation programme can have a profound impact on the relational aspects of care and the development of a collaborative culture. The RC framework—shared knowledge, shared goals and mutual respect in the context of high-quality communication—can provide a common language for simulation educators to design and debrief simulation exercises that aim to have a translational impact. Simulation educators should be deliberate about the foundational team relationship and organisational culture outcomes of the simulation programmes they develop.
